# Case Report: Flagellate eruption of adult-onset still disease in an Afro-Caribbean woman: highlighting atypical cutaneous disease in richly pigmented skin

**DOI:** 10.3389/fimmu.2026.1734042

**Published:** 2026-03-03

**Authors:** Ceylon Gomes, Xaneile Facey, Danielle Morris, Jonathan D. Ho

**Affiliations:** 1 Divison of Dermatology, Department of Medicine, The University of the West Indies, Kingston, Jamaica; 2 Department of Pathology, The University of the West Indies, Kingston, Jamaica

**Keywords:** adult-onset still disease, atypical still disease, black skin, flagellate, persistent pruritic papules and plaques, skin of color

## Abstract

Adult-onset Still disease (AOSD) is an uncommon autoinflammatory disorder characterized by high spiking fevers, arthralgia, and, classically a transient, salmon-colored rash. Atypical cutaneous variants, such as persistent erythematous papules and plaques often arranged in a flagellate pattern, are increasingly reported; however, few cases illustrate this morphology in individuals with richly pigmented skin. We report a 70-year-old Afro-Caribbean woman (Fitzpatrick skin type VI) with persistent high-grade fevers, polyarthritis and diarrhea of unknown origin which persisted despite multiple courses of antibiotics. Laboratory evaluation revealed leukocytosis and transaminitis. Dermatology was consulted for a cutaneous eruption present for three weeks prior to hospitalization. Examination revealed subtle red-brown papules, plaques and patches on the trunk and extremities, some in a flagellate arrangement. Skin biopsy demonstrated hyperkeratosis, individually necrotic keratinocytes in the upper epidermis and a perivascular neutrophilic infiltrate characteristic of the atypical eruption of AOSD. Further blood work confirmed marked hyperferritinemia. Treatment with systemic and topical corticosteroids resulted in improvement. This case underscores the importance of recognizing atypical cutaneous variants of AOSD in persons with richly pigmented skin to prevent diagnostic delay and improve clinical outcomes.

## Introduction

Adult-onset Still Disease (AOSD) is an uncommon autoinflammatory disorder that classically presents with recurrent episodes of high spiking fevers, arthralgias and/or myalgias and an evanescent, urticarial, non-pruritic salmon colored rash (1). Flagellate patterning of persistent papules and plaques is an atypical presentation that is increasingly frequently reported. Most of these presentations however are shown in lightly pigmented Fitzpatrick skin types I-III and there are limited reports on this presentation in persons with richly pigmented skin. We report such a case and highlight examples of similar presentations in Afro-Caribbean persons with richly pigmented skin types.

## Case description

A 70-year-old Afro-Caribbean female with no known comorbidities and taking no medications presented to the emergency room with a two-week history of polyarthritis, lethargy, recurrent fevers, watery diarrhea, decreased appetite, and sore throat. Joint pains involved the wrists, elbows, knees and shoulders. Patient sought medical attention prior to presentation and was treated for a urinary tract infection with amoxicillin clavulanate, but this was discontinued due to perceived worsening of her gastrointestinal symptoms. Examination revealed an elderly female in pain. No synovitis, joint swelling, hepatosplenomegaly or lymphadenopathy was noted. The patient was admitted for further evaluation.

Laboratory investigations demonstrated marked leukocytosis (27.3 x10^9^/L) with neutrophilia (90% neutrophils; absolute neutrophil count [ANC] 24,550 cells/μL), elevated transaminases, hypoalbuminemia, elevated erythrocyte sedimentation rate (90 mm/hr) and c-reactive protein (28.56mg/dL). Peripheral smears revealed toxic granulation and vacuolation of neutrophils. The remainder of the complete blood count, electrolytes, blood urea nitrogen, creatinine, creatinine kinase and complement levels (C3, C4) were normal. The patient was assessed as an acute viral illness with a superimposed bacterial infection in light of the striking neutrophilia and commenced on intravenous ceftriaxone. Two blood cultures were negative and one grew coagulase negative staphylococcus in one of two bottles consistent with contamination. Urine culture was negative for pathogens. Near daily febrile episodes up to 40°C, continued to occur, primarily in the evenings (quotidian pattern), though occasional morning spikes (double-quotidian pattern) were also noted. Onset of diarrhea prompted stool testing. No ova cysts or parasites were identified. A non-toxin producing *Clostridium difficile* was isolated. Despite lack of toxin production, given unresolved gastrointestinal symptoms, oral vancomycin was commenced, with no improvement. Viral serology including human immunodeficiency, dengue, cytomegalovirus, Epstein Barr virus, human T lymphotropic virus 1/2 were negative. Polymerase chain reaction for SARS−CoV−2, influenza A/B were also negative. Venereal Disease Research Laboratory serology was non-reactive. Urinalysis was normal without casts, blood or proteinuria. Continued pyrexia, diarrhea and new-onset cough prompted broadening of antimicrobial coverage to intravenous piperacillin-tazobactam with little improvement.

**Figure 1:**
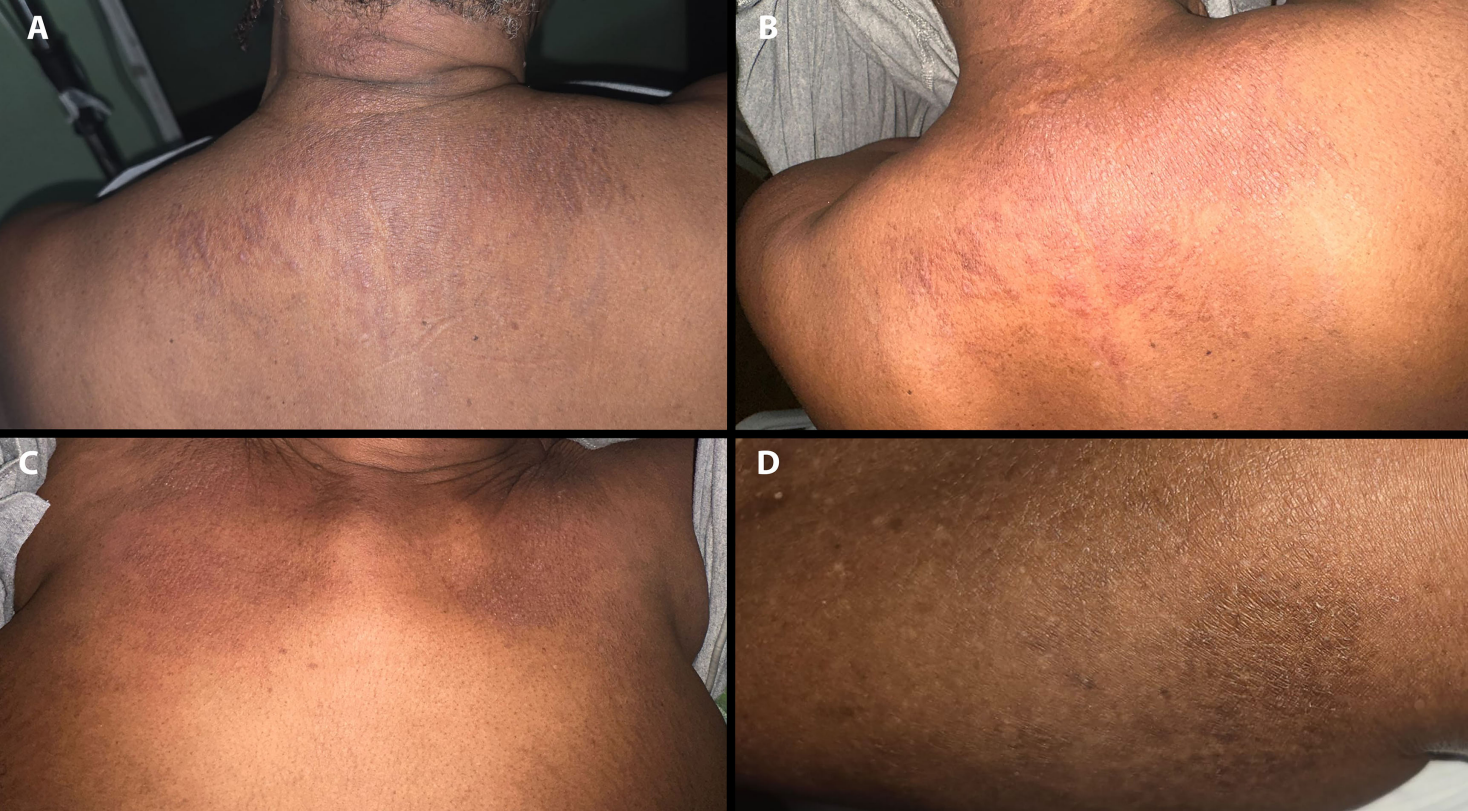
Atypical eruption of adult-onset still disease in an Afro-Caribbean female. Brown hyperpigmented papules in a downward flagellate (linearized) arrangement on the upper back and posterior neck **(A)**. There is subtle background violaceous erythema **(A)**. Flash photography hides the brown and papular component but highlights red-erythema **(B)**. Relatively symmetric brown hyperpigmented, slightly scaly patches and thin plaques are present on the chest **(C)**. Subtle hyperpigmentation is present on the calf **(D)**.

**Figure 2:**
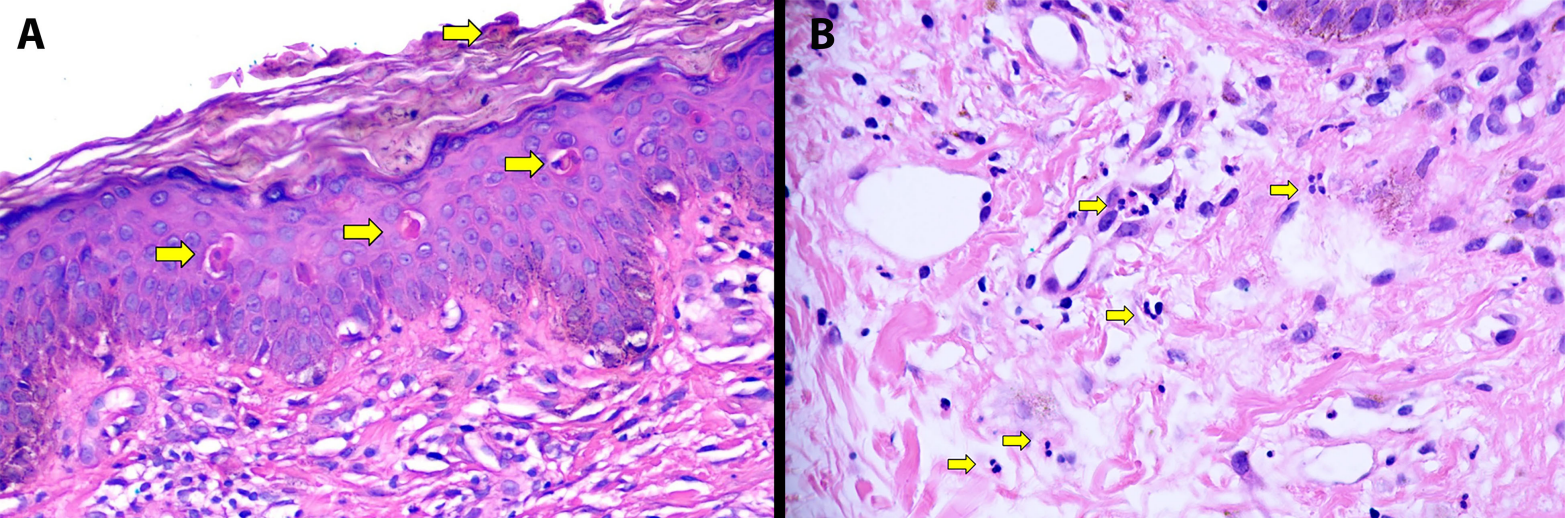
Characteristic histopathology of flagellate eruption in adult-onset still disease. Hyperkeratosis and individually necrotic keratinocytes present in the stratum corneum and mid-to-upper epidermis (**(A)** arrows) with relative lack of basal apoptosis is seen in the majority of atypical eruptions. Perivascular and interstitial neutrophils (**(B)** arrows) are also present bridging the histopathology of evanescent and persistent eruptions.

Two weeks into hospitalization a rash was noted and Dermatology consulted. On further discussion, the patient reported that the rash started 3 weeks before admission, persisted throughout her hospital stay and endorsed significant pruritus. Her room was relatively dimly lit and the eruption was not previously appreciated by the managing team. Skin examination revealed a patient with Fitzpatrick skin type VI and red-brown slightly hyperkeratotic papules on the trunk (**Figures 1A, B**). On the chest the lesions formed thin plaques (**Figure 1C**). On the upper back downward linearization of the papules imparted a subtle flagellate (whip-like) pattern (**Figure 1A**). Erythema was significantly more visible with enhanced lighting (**Figure 1B**). Similar lesions were present on the knees, calves and abdomen (**Figure 1D**). No urticarial lesions were present. Given the clinical setting, the persistent pruritic flagellate eruption of AOSD was suspected. Although the upper back distribution and somewhat violaceous erythema was reminiscent of a shawl sign in dermatomyositis, no central face erythema, eyelid edema/erythema, Gottron papules, holster sign or nail fold telangiectasia were present. No mucosal, scalp or nail involvement was noted. No lymphadenopathy or hepatosplenomegaly was palpated. Two 4-mm punch biopsy skin samples were performed from the posterior neck and the upper back. Both specimens exhibited similar features of hyperkeratosis, apoptotic keratinocytes high in the epidermis in the absence of significant basal vacuolation and a superficial perivascular lymphocytic infiltrate with numerous neutrophils (**Figure 2**). No interface dermatitis, increased dermal mucin or superficial and deep perivascular lymphocytic infiltrate characteristic of autoimmune connective tissue disease were identified. PAS stain failed to reveal a thickened basement membrane zone. The distinctive histopathologic and clinical findings fulfilling the Yamaguchi criteria were diagnostic of AOSD with persistent, flagellate pruritic papules and plaques (**Table 1**) (2). We requested a ferritin level which was markedly elevated (28,132 ng/mL). Glycosylated ferritin evaluation was not available. An antinuclear antibody titer (ANA) was weakly positive (1:40). Antibodies against double stranded DNA, Smith, Ro, La and U1RNP were negative. RF was also negative. Notably, repeat ANA titer (4 weeks after the initial test results) was negative. Other diagnostic and prognosticating biomarkers (e.g. interleukin-18, interleukin-1β and calprotectin) were unavailable. Given the very high ferritin levels, impending hemophagocytic lymphohistiocytosis was considered. Lack of cytopenias, normal triglyceride levels and lack of hepatosplenomegaly on abdominal ultrasound made this complication unlikely.

**Table 1:** Yamaguchi et al. criteria (2) and reported atypical rashes in adult-onset still disease beyond flagellate eruptions (3–14).

Criteria*	Case	Reported morphologic variants
Major		Dermatomyositis-like Prurigo pigmentosa-like Follicular accentuation Erythema Multiforme-like Fixed eruptions Erythroderma Purpuric lesions Acral pustular eruption Nasal perforation Oral pigmentation
Fever ≥39 °C for ≥1 week	+	
Arthralgia/arthritis ≥2 weeks	+	
Typical rash	+^**^	
Leukocytosis ≥10,000/mm^3^ [≥80% neutrophilia]	+	
Minor		
Sore throat	+	
Lymphadenopathy	–	
Hepatomegaly/splenomegaly	–	
Deranged liver function tests	+	
Negative ANA/RF	–	
Exclusion of:		
Malignancy (primarily lymphoma) Other rheumatic diseases (primarily Rheumatoid vasculitis and PAN) Infections (primarily sepsis and IM)	+	

*Requires 5 positives, two of which must be major criteria.

**While typical rash initially referred to the evanescent rash, consistent and increasing reporting of flagellate eruptions confirms its characteristic presence in adult-onset Still disease.

ANA, antinuclear antibody; IM, infectious mononucleosis; PAN, polyarteritis nodosa; RF, rheumatoid factor.

**Figure 3:**
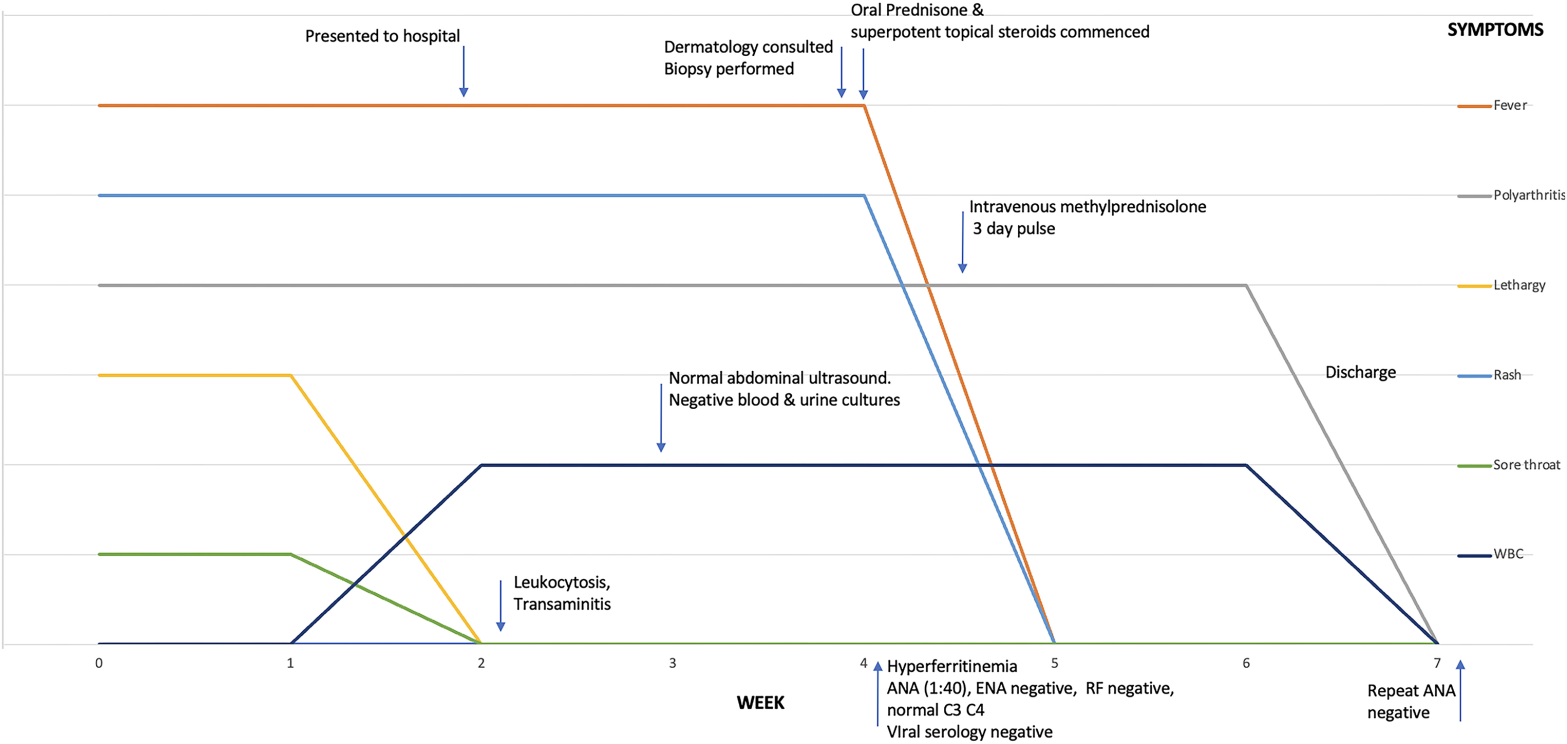
Graphical timeline of key clinical features, diagnostics and therapeutic intervention. ANA, antinuclear antibodies; ENA, extractable nuclear antigens; RF, rheumatoid factor.

While methotrexate tocilizumab and/or IL-1 inhibitors were considered, due to need for rapid control of symptoms and delays in immediate access to biologics (insurance authorization, tuberculosis screening and hospital formulary restrictions), systemic steroids at 1 mg/kg (80 mg daily) and superpotent topical steroids (clobetasol propionate 0.05% ointment) were commenced with defervescence and significant improvement in joint pain, diarrhea and pruritus within 72 hours. White cell counts improved as did her transaminitis. On day four however, the patient had return of significant joint pain and received intravenous methylprednisolone 500 mg for three days. This resulted in resolution of recrudescent arthralgia. The patient was discharged on 80 mg of prednisone and referred to rheumatology for further management and commencement of steroid sparing/biologic agent. Up until the time of writing however (6 weeks after discharge), the patient has not attended follow up appointments due to a long commute to the hospital. We have however communicated with the patient via telephone and she endorses complete resolution of joint pains, rash and fever. We have advised her to keep her clinic appointments for appropriate management of her systemic steroid and selection of an appropriate steroid sparer. **Figure 3** summarizes the timeline of key clinical, diagnostic and therapeutic features.

## Discussion

AOSD is a multisystem inflammatory disease of unknown etiology, typically characterized by spiking fevers, an evanescent rash, and joint pain (1, 15). The classical rash is salmon-pink, transient and urticarial; however atypical cutaneous presentations with persistent lesions are increasingly reported. Despite being a diagnosis of exclusion, early recognition of the cutaneous manifestations can significantly alter the treatment course and shorten time to diagnosis. This was demonstrated in our case, where the patient was admitted and treated with multiple antibiotics until referral for the rash (which was present for weeks before presentation) facilitated diagnosis. Like many inflammatory dermatoses, the appearance of the cutaneous manifestations of AOSD specifically the erythematous component is more difficult to identify in more richly pigmented persons (16). Despite increased recognition of non-urticarial variants, there is relatively poor representation of atypical eruptions in the most pigmented races and particularly Black patients. Using search strategy “(atypical) OR (flagellate)) OR (persistent)) OR (papules)) OR (plaques)) AND (adult onset still) in PubMed/MEDLINE database, we identified 59 cases of atypical eruptions of AOSD with accompanying images. 15 cases in medium to dark skin types were identified (3, 4, 17–29). The majority of these were medium pigmented persons of South East Asian or Indian descent with only four representations in Black patients (22, 26, 27, 29). In these cases, the primary morphology was a linearizing or flagellate (whip-like) eruption with brown hyperpigmentation or maroon to violaceous color, sometimes with scale. Our patient presented with a brown, subtly violaceous and flagellate papular eruption on the upper back. Though initially inapparent, with additional lighting, red-erythema was also identified (**Figure 1A**). This appearance, together with brown, non-erythematous patches and thin plaques present on the chest and limbs (**Figure 1B**), highlight the importance of recognizing hyperpigmented-predominant morphologies. Examining patients in adequate and frequently auxiliary light is critical for recognition of erythema in high Fitzpatrick skin types and this applies to both the typical urticarial and atypical eruptions (30). A helpful maneuver when assessing apparently hyperpigmented eruptions in richly pigmented skin involves pressing the pigmented areas with a finger or glass microscopic slide (diascopy). If the “hyperpigmentation” is primarily due to vasodilation-related erythema, the area will blanch and gradually regain color on removal of pressure. Importantly, this maneuver will not help with purpuric erythema due erythrocyte extravasation. While persistent pruritic flagellate/linear papules and plaques is the most commonly described atypical pattern, multiple morphologies including prurigo pigmentosa-like, cutaneous amyloidosis-like and dermatomyositis-like presentations have been reported (31). Variant atypical presentations may be seen across all skin tones. The key differences include subtle/absent red-erythema, propensity for violaceous-erythema and significant hyperpigmentation. Recognition of the widening variety of presentations helps to avoid misclassification and diagnostic delay. Early diagnosis may be especially important for those with atypical eruptions which have been reported in association with more persistent and severe disease including pulmonary involvement, serositis and cardiac disease (32). This was true in our patient who had colitis, high leukocytosis, hepatic and pulmonary involvement all responsive once immunosuppression was initiated.

Helpfully, the histopathologic findings are similar and characteristic across atypical presentations. As in this case, up to 96% of flagellate eruptions and ~80% of other atypical rashes demonstrate necrotic keratinocytes in the mid to upper, sometimes acanthotic, epidermis (“high” apoptosis) and a perivascular lymphocytic infiltrate with neutrophils (31–33). Other entities frequently exhibiting high apoptotic keratinocytes include photolichenoid eruptions, fixed sunlight eruptions, nutritional deficiencies, glucagonoma syndrome, and erythema multiforme (34). Most of these entities lack the perivascular neutrophilic infiltrate, present without flagellate eruptions, have distinct morphologies (e.g. target lesions in erythema multiforme or flexural erosive disease in nutritional deficiencies) and lack systemic features of severe autoinflammation (34). Basal layer vacuolation is either absent or mild helping to differentiate causes of interface dermatitis of autoimmune connective tissue disease (32, 33). Epidermal involvement (hyperkeratosis, acanthosis and high apoptotic keratinocytes) is the major difference histopathologic between the atypical and classical AOSD, with the latter demonstrating only the perivascular neutrophilic infiltrate (32–34). While the histology of the evanescent urticarial lesions of AOSD generates a relatively broad differential diagnosis (neutrophilic urticaria, neutrophilic urticarial dermatoses, periodic fever syndromes and autoimmunity-related neutrophilic dermatosis), the combination with characteristic epidermal findings in the correct clinical setting is strongly supportive, even if not entirely diagnostic (31–34). Familiarity with these findings is crucial to distinguish AOSD from mimickers.

Differentiating the rashes of AOSD from lookalikes is critical. The clinical differential diagnosis for a flagellate eruption includes bleomycin and other drug-induced pigmentation, shitake mushroom dermatitis, scabietic infestation, marine stings, religious flagellation and dermatomyositis (35). Like many of these entities, pruritus with isomorphic phenomenon (Koebnerization) likely contributes to the linear appearance in AOSD (36). Clinical history easily excludes the majority but distinguishing dermatomyositis from AOSD deserves special attention. Like in our patient, distribution of the eruption on the upper back with hyperpigmentation and violaceous erythema may mimic a shawl sign. To complicate matters, dermatomyositis may present primarily with flagellate eruptions (zebra stripe dermatomyositis) and AOSD may present with a heliotrope rash and articular erythema (5, 36, 37). Key distinguishing features include marked neutrophilia in AOSD and presence of characteristic cutaneous histopathology as described above (6–8). Although dermatomyositis and other autoimmune connective tissue diseases may have elevated ferritin levels, massive hyperferritinemia (>10,000 ng/mL) is exceptional and strongly suggests autoinflammatory rather than autoimmune disease (38). Importantly, this patient’s positive ANA could lead to a misdiagnosis of autoimmune connective tissue disease (AICTD). A number of features however argue for AOSD and against AICTD, particularly systemic lupus erythematosus (SLE). Like dermatomyositis, striking neutrophilia and massive hyperferritinemia are not typical of SLE. Unlike dermatomyositis, a flagellate rash is not compatible with the well-defined eruptions of cutaneous lupus erythematosus (CLE). Additionally, the histopathology CLE reveals classical interface dermatitis often with superficial and deep perivascular lymphocytic inflammation and increased dermal mucin rather than the characteristic pattern in AOSD (39). Although neutrophils may be present, they exist in select subtypes with clearly identifiable morphologies including bullous SLE, neutrophilic urticarial dermatoses and amicrobial pustulosis of the folds (40). This patient also lacked other features of SLE including renal disease or cytopenias. Importantly, repeat ANA and antibodies against double-stranded DNA, extractable nuclear antigens and complement levels were negative/within normal ranges. Finally, ANA positivity has been reported in patients with AOSD and patients with defined AICTD including SLE and Sjogren syndrome may develop AOSD (41–44). Other AICTDs initially considered including rheumatoid arthritis were not supported by the patients negative RF, morphology and histopathology of the eruption and the laboratory findings suggesting marked autoinflammation rather than autoimmunity. Similar anomalies involving the Yamaguchi exclusion criteria (malignancy, rheumatic disease and infections) are well established as AOSD associated with delayed onset malignancies and in the setting of viral and bacterial infections are documented with some frequency (45–47). Careful attention to rash morphology, laboratory features of massive systemic inflammation and histopathology help correctly diagnose AOSD even in the presence of “confounders”. As described above however, a growing list of morphologies have been reported and a high index of suspicion for AOSD should be maintained for any patient presenting with spiking fevers, signs of systemic inflammation and a rash. **Table 1** highlights unusual cutaneous presentations of AOSD beyond flagellate eruptions (3–14). Algorithmically, AOSD should be considered in any adult with fever of known origin and a rash. If the rash is clinically and histopathologically typical of a known entity with supporting ancillary investigations (e.g. malar erythema in a young woman with positive ANA and dsDNA), AOSD is unlikely. However, if the eruption is urticarial, flagellate, non-specific or even resembles another well-defined entity *and* histopathology reveals a perivascular neutrophilic infiltrate with/without apoptotic keratinocytes high in the epidermis, AOSD should be seriously considered and supporting historical and laboratory findings sought. Exclusion criteria as described in various diagnostic criteria should be adhered to, but recognition of caveats and nuances as discussed are critical to avoid misclassification. Limitations include lack of complete follow up data as the patient has yet to keep their follow-up appointment and unavailability of more advanced prognosticating and diagnostic biomarkers.

## Conclusion

In summary, we highlight the importance of early recognition of the atypical eruptions of AOSD. While this presentation is not novel, the potential subtlety and color variations in very richly pigmented skin types are relatively infrequently reported. Lack of recognition of a rash resulted in delayed diagnosis for our patient despite hospitalization. Despite the important consideration of AICTD and other mimickers, familiarity with its protean cutaneous appearance, laboratory and histopathologic features are often sufficient to correctly distinguish the atypical eruptions of AOSD from mimickers. This case increases the visual representation of a rare entity in persons with richly pigmented skin.

## Data Availability

The original contributions presented in the study are included in the article/supplementary material. Further inquiries can be directed to the corresponding author.
